# Progesterone Acts via the Nuclear Glucocorticoid Receptor to Suppress IL-1β-Induced COX-2 Expression in Human Term Myometrial Cells

**DOI:** 10.1371/journal.pone.0050167

**Published:** 2012-11-28

**Authors:** Kaiyu Lei, Li Chen, Ektoras X. Georgiou, Suren R. Sooranna, Shirin Khanjani, Jan J. Brosens, Phillip R. Bennett, Mark R. Johnson

**Affiliations:** 1 Chelsea and Westminster Hospital, London, United Kingdom; 2 Institute of Reproductive and Developmental Biology, London, United Kingdom; 3 Department of Obstetrics and Gynecology, Third Military Medical University Southwest Hospital, Chongqing, P. R. China; Fudan University, China

## Abstract

Progesterone is widely used to prolong gestation in women at risk of preterm labour (PTL), and acts at least in part via the inhibition of inflammatory cytokine-induced prostaglandin synthesis. This study investigates the mechanisms responsible for this inhibition in human myometrial cells. We used reporter constructs to demonstrate that interleukin 1beta (IL-1β) inhibits progesterone driven PRE activation via p65 activation and that IL-1β reduced progesterone driven gene expression (FKBP5). Conversely, we found that the activity of a p65-driven NFκB reporter construct was reduced by overexpression of progesterone receptor B (PRB) alone and that this was enhanced by the addition of MPA and that both MPA and progesterone suppressed IL-1β-driven cyclo-oxygenase-2 (COX-2) expression. We found that over-expressed Halo-tagged PRB, but not PRA, bound to p65 and that in IL-1β-treated cells, with no overexpression of either PR or p65, activated p65 bound to PR. However, we found that the ability of MPA to repress IL-1β-driven COX-2 expression was not enhanced by overexpression of either PRB or PRA and that although the combined PR and GR antagonist Ru486 blocked the effects of progesterone and MPA, the specific PR antagonist, Org31710, did not, suggesting that progesterone and MPA act via GR and not PR. Knockdown using siRNA confirmed that both MPA and progesterone acted via GR and not PR or AR to repress IL-1β-driven COX-2 expression. We conclude that progesterone acts via GR to repress IL-1β-driven COX-2 activation and that although the interaction between p65 and PRB may be involved in the repression of progesterone driven gene expression it does not seem to be responsible for progesterone repression of IL-1β-induced COX-2 expression.

## Introduction

Progesterone is widely used in several different formulations to reduce the risk of preterm labour in high-risk singleton pregnancies [1]–[4]. However, the mechanisms underpinning this effect are unclear. The precipitous fall in systemic progesterone levels that occurs before the onset of labour in virtually all non-primates does not occur in the human. However, the seminal work of Csapo [5], who showed that progesterone was essential for the maintenance of early pregnancy, and the observation of Frydman et al, that mifepristone (Ru486), the progesterone (and glucocorticoid) antagonist, can be used to induce labour [6], suggest that progesterone does play an important role in the maintenance of pregnancy in the human too. Animal studies suggest that progesterone acts predominantly to allow the uterus to tolerate stretch; in its absence uterine distension (with a pregnancy or artificially) up-regulates the expression of prolabour factors such as oxytocin receptor [7] and connexin-43 [8], stimulating the onset of labour. Progesterone does not seem to have a similar role in the human since randomised studies of progesterone administration in multiple pregnancies failed to show any prolongation of pregnancy [9], [10] and in vitro studies failed to show any effect on stretch-induced gene expression [11]. This suggests that in the human progesterone acts through different pathways to delay the onset of labour.

A mutual repression has been shown to exist between NFκB and PR in reproductive tissues, which may determine the timing of the onset of labour [12]. Initial work reported that the trans-repression between PR and NFκB occurs independent of PR isoform, reporter construct or cell type and, since PR and RelA interacted in vitro, suggested that the mutual repression was due to a direct interaction between the proteins [13]. This work was performed in Hela, Cos and the human breast tumour, T47D, cell lines, with over expression of PRB, PRA and Rel-A and using PRE, MMTV and NFκB reporter constructs [13]. Later studies by our group, in amnion cells, showed that IL-1β was able to repress progesterone-activation of a progesterone reporter construct and that overexpression of PR repressed the activity of an NFκB reporter construct [14]. Consistent with this, progesterone suppressed the IL-1β-induced expression of COX-2 mRNA in immortalized human fundal myometrial cells [15]. ChIP studies showed that the IL-1β-induced increase in p65 binding to both proximal and distal NFκB binding elements of the *COX-2* promoter was reduced by progesterone [15]. They also found that progesterone induced the expression of IκB, which binds to p65 in the cytoplasm, and concluded that this was the likely explanation for progesterone repression of IL-1β activity [15]. These and our earlier data involve the use of over-expression of PR and/or p65 and also the use of cell lines. Consequently, uncertainty remains as to the true nature of the relationship between progesterone, its receptors and NFκB in myometrial cells.

In this paper we have re-examined the evidence in support of the existence of a mutual repression between PR and NFκB and investigated potential alternative explanations for the inhibition of inflammatory cytokine driven COX-2 expression by progesterone. We have used progesterone and NFκB-responsive genes to assess the degree of mutual repression, complimented by inhibitor and knock down studies, in addition to reporter constructs and over-expression of PR and NFκB.

**Table 1:** ON-TARGET plus SMART pool siRNA sequences.

Name	Target Sequences
p65	GGAUUGAGGAGAAACGUAA CCCACGAGCUUGUAGGAAA GGCUAUAACUCGCCUAGUG CCACACAACUGAGCCCAUG
PR	GGACGUGGAGGGCGCAUAU GAGAUGAGGUCAAGCUACA ACAUAUUGAUGACCAGAUA GCACCUGAUCUAAUACUAA
GR	GAACUUCCCUGGUCGAACA GGAAACAGACUUAAAGCUU UGACAAAACUCUUGGAUUC GCAUGUACGACCAAUGUAA
AR	GAGCGUGGACUUUCCGGAA UCAAGGAACUCGAUCGUAU CGAGAGAGCUGCAUCAGUU CAGAAAUGAUUGCACUAUU
HSD11β1	GAAGAGAGAUGGCUUAUCA GAAAGUGGCUUAUCCAAUG GAAGAAGUGUAUUAUGACA UCAACGAGCUAUAAUAUGG

## Experimental Procedures

### Tissue Specimens

Myometrial biopsies (0.5×0.5×0.5 cm^3^) of term human myometrium were collected at the time of elective caesarean section from the upper margin of the incision made in the lower segment of the uterus from women not in labour. Samples were then put into Dulbecco’s modified Eagle’s Medium (DMEM, Invitrogen, Paisley, PA4 9RF) medium containing L-glutamine and 100 mU/mL penicillin and 100 µg/mL streptomycin and were stored at 4°C for no more than 3 h prior to cell preparation for culture.

### Ethics Statement

The Riverside Ethics Committee approved the study. All specimens were obtained after fully informed, written patient consent.

### Primary Cell Culture

Primary human USMCs were isolated using a mixture of collagenases [1 mg/ml of collagenase 1A and 1 mg/ml of collagenase XI (Sigma)] and cultured in DMEM medium containing phenol red 7.5% fetal calf serum, L-glutamine and 100 mU/mL penicillin and 100 µg/mL streptomycin in an atmosphere of 5% CO_2_∶ 95% air at 37°C. Myometrial cells grown in this manner have previously been characterized [16]. Cells from passage 1 to 4 [progesterone receptor levels are maintained with passaging (unpublished observation)] were trypsinised in 0.25% trypsin containing 0.02% EDTA and cultured in 24-well, 6-well culture plates or flasks depending on the requirement. In some cases at the end of the specified time, medium was removed and cells were frozen at −80°C for the extraction of RNA, protein or the luciferase assay. In other cases, such as co-immunoprecipitation (co-IP), cells were harvested and processed directly after treatment.

Before treating the cells with different stimuli, old medium was removed and replaced with 2 mL of fresh-stripped medium (1% Charcoal and Dextran-stripped fetal calf serum, supplemented with L-glutamine, 100 mU/ml penicillin and 100 g/ml streptomycin) overnight. In some cases, cells were pre-incubated with 1 µM RU486 (GR/PR antagonist, Sigma-Aldrich Company Ltd., Dorset, SP8 4XT), 1 µM Org31710 (PR selective antagonist, N.V. Organon) for 2 h prior to other stimuli, such as IL-1β (5 ng/mL), MPA (1 µM), progesterone (10 µM) and Dex (1 µM), either alone or in combination. Ethanol was used as vehicle. The concentration of MPA and P4 used in this study has been opitimised previously (Fig. S1) and to be physiologically relevant [17], [18].

**Table 2:** Primer pair sequences with gene accession numbers.

Name	Forward (F) and Reverse (R) primer sequence (5′–3′)	Genebank/EMBL Accession no.
COX-2	F: tgtgcaacacttgagtggct	AY151286
	R: actttctgtactgcgggtgg	
FKBP5	F: tccctcgaatgcaactctct	NM_001145775
	R: gccacatctctgcagtcaaa	
GAPDH	F: tgatgacatcaagaaggtggtgaag	BC014085
	R: tccttggaggccatgtaggccat	
HSD11β1	F: accttcgcagagcaatttgt	NM_005525
	R: gccagagaggagacgacaac	

### Luciferase Assays

Cells were cultured in 24-well culture plates to about 80% confluence. Expression constructs (pSG5 and pSG5-p65 were kind gift from Dr. John White, Imperial College London, UK; pSG5-PRB was given by Dr. Pierre Chambon, Institut de Genetique et de Biologie Moleculaire et Cellulaire, Strasbourg, France) and reporter vector [PRE was kindly provided by Dr. Birgit Gellersen, Hamburg, Germany; pNFκB-Luc (Clontech)] were co-transfected by Gene-Juice transfection reagent (Merck Chemicals Ltd.) at concentrations of 300 ng/well and SV40-Renilla vector pRL-SV40 (Promega, Chilworth, Southampton, SO16 7NS) was used as an inner control for transfection efficiency at concentrations of 100 ng/well. The empty expression vector pSG5 was included as filler construct so that the total amount of transfected DNA per well was constant. Cells were treated with the specific stimulus or vehicle at 24 h post transfection. After another 24 h, firefly luciferase activity was measured by using a dual firefly/renilla luciferase assay (Luclite, PerkinElmer LAS UK Ltd., Seer Green, HP9 2FX) and Coelenterazine (Merck Chemicals Ltd.). Results of luciferase activity was first normalized to the level of Renilla luciferase activity and then calculated as fold induction relative to either expression of vehicle-treated group, or the control empty expression vector.

**Figure 1:**
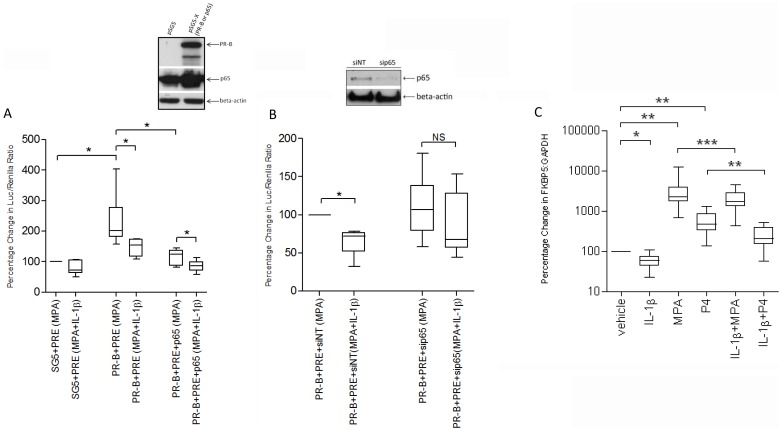
IL-1β represses progesterone action via NFκB. **A,** Myometrial cells were transiently co-transfected with a progesterone response element (PRE), with or without progesterone receptor B (PRB) and p65. SG5 was used as control. MPA and IL-1β were added 24 h after transfection and the cells were incubated for another 24 h before luciferase assay. **B,** Myometrial cells were transfected with either siRNA for p65 (sip65) or a non-targeting siRNA (siNT) as control. After 72 h, cells were co-transfected with PRB and PRE. MPA and IL-1β were added at day 4 post-transfection and cells were then incubated for another 24 h before luciferase assay. **C,** Myometrial cells were exposed to different stimuli, IL-1β, MPA and progesterone, either alone or in combination. mRNA was then extracted, and the FKBP5 mRNA levels were measured using qPCR. Data are expressed as median, 25^th^ and 75^th^ percentiles and range, and were analysed using Wilcoxon matched pairs test. *indicates a significant difference of *p*<0.05, **, of *P*<0.01, and ***, of *P*<0.001; NS indicates no statistic significant difference (n = 6–12).

**Figure 2:**
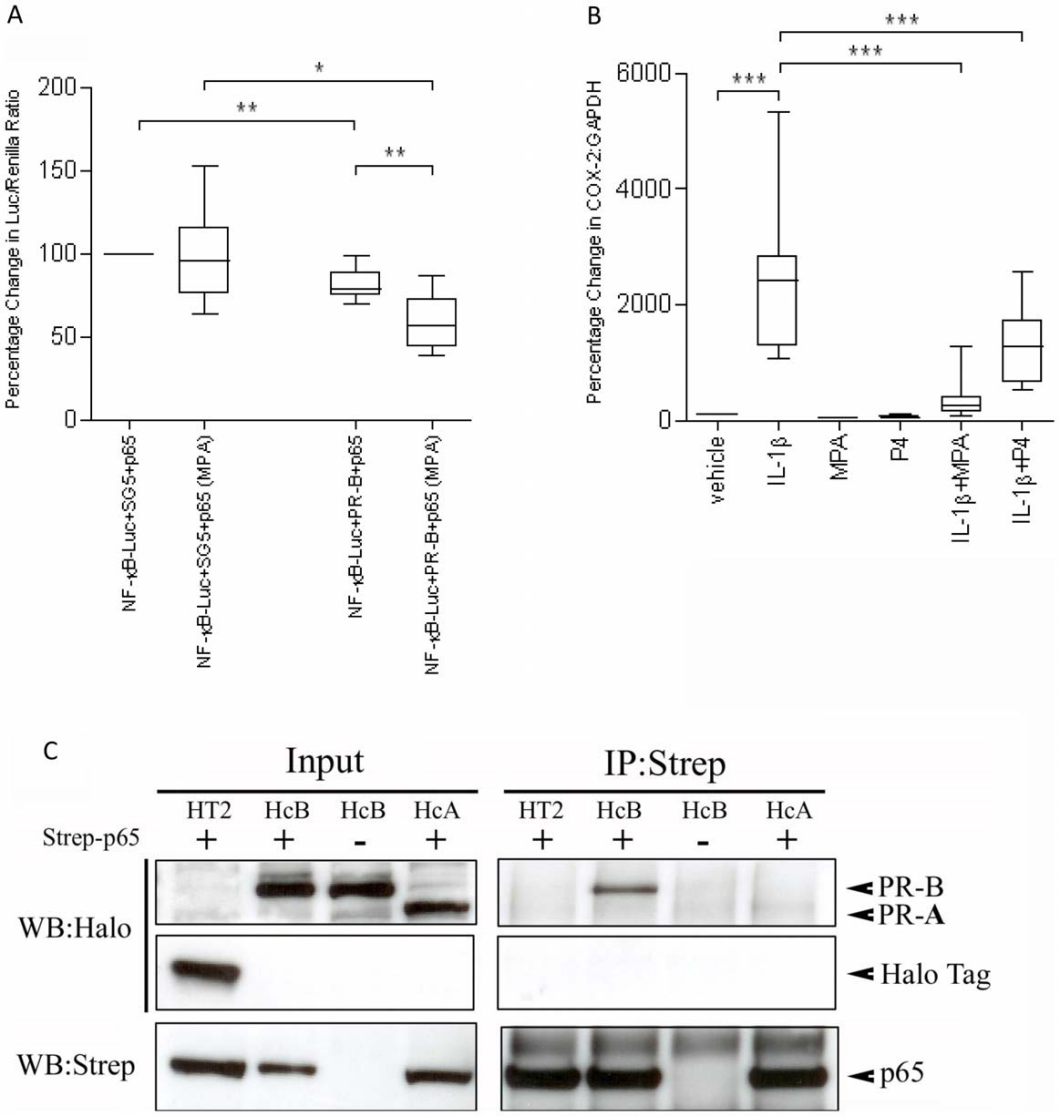
PRB inhibits NFκB activity. **A,** Myometrial cells were transiently co-transfected with a NFκB luciferase construct (NFκB-Luc) and p65, with or without PRB. SG5 was used as control. MPA was added 24 h after transfection and the cells were incubated for another 24 h before luciferase assay. Data are expressed as median, 25^th^ and 75^th^ percentiles and range, and were analysed using paired *t* test. *indicates a significant difference of *p*<0.05 and **, of *P*<0.01 (n = 6). **B,** Myometrial cells were exposed to different stimuli, IL-1β, MPA and progesterone, either alone or in combination. mRNA was then extracted, and the COX-2 mRNA levels were measured using qPCR. Data are expressed as median, 25^th^ and 75^th^ percentiles and range, and were analysed using paired *t* test. ***indicates a significant difference of *p*<0.001 (n = 12). **C,** Strep-tagged p65 (Strep-p65) and Halo-tagged PRA (HcA) or PRB (HcB) were transfected into myometrial cells as indicated. Parental halo-tag construct (HT2) was used as control. Cell lysates were then immunoprecipitated using anti-Strep antibody. The immunoprecipitates were examined by Western blotting using anti-Halo antibody. Input represented 10% of cell lysates used in the co-IP experiment.

### Gene Overexpression and Silencing

Both gene expression constructs and siRNAs (Table 1) were transfected using the Amaxa Nucleofector technology (Lonza Sales AG, Muenchensteinerstrasse 38, Basel, CH-4002). Cells were harvested by trypsinization as described above. Approximately 1×10^6^ cells were resuspended in 100 µl room-temperature Nucleofector Solution and mixed with either 2 µg of DNA constructs [pHT2 was kind gift from Dr. Mark Christian, Imperial College London, UK; pFC14A-PRA and pFC14A-PRB were cloned by inserting the ORF of PRA or PRB into the pFC14A vector between SgfI and EcoICRI sites; pQE-p65 was kindly provided by Dr. Shirin Khanjani, Imperial College London, UK; pSG5-PRA and pSG5-PRB were given by Dr. Pierre Chambon, Institut de Genetique et de Biologie Moleculaire et Cellulaire, Strasbourg, France; pSG5-GR was kindly given by Dr. Charlotte Bevan, Imperial College London, UK] or different amount of siGENOME SMARTpool siRNA (30 pmol of p65, PR, GR, HSD11β1; 90 pmol of AR; Thermo Fisher Scientific, Abgene Ltd., Epsom KT19 9AP). The cell/siRNA suspension was then transferred into certified cuvette and electroporated in the Nucleofector Cuvette Holder with the Nucleofector Program A-033. Immediately after electroporation, cells were suspended with 500 µl pre-warmed culture medium and gently transferred into 6-well culture plates. The cells were then incubated in an atmosphere of 5% CO_2_, 95% air at 37°C until analysis.

### Quantitative RT-PCR

Total RNA was extracted and purified from myometrial cells grown in 6-well culture plates using RNAeasy mini kit (Qiagen Ltd., Crawley, West Sussex, RH10 9AX). After quantification 1.5 µg was reverse transcribed with oligo dT random primers using MuLV reverse transcriptase (Applied Biosystems Ltd., Warrington, Cheshire, WA3 7PB). Primer sets (Table 2) were designed and obtained from Invitrogen. Quantitative PCR was performed in the presence of SYBR Green (Roche Diagnostics Ltd., Burgess Hill, West Sussex, RH15 9RY), and amplicon yield was monitored during cycling in a RotorGene Sequence Detector (Corbett Research Ltd., Mortlake, Sydney, Australia) that continually measures fluorescence caused by the binding of the dye to double-stranded DNA. Pre-PCR cycle was 10 min at 95°C followed by up to 45 cycles of 95°C for 20 sec, 58–60°C for 20 sec and 72°C for 20 sec followed by an extension at 72°C for 15 sec. The final procedure involves a melt over the temperature range of 72–99°C rising by 1degree steps with a wait for 15 sec on the first step followed by a wait of 5 sec for each subsequent step. The cycle at which the fluorescence reached a preset threshold (cycle threshold) was used for quantitative analyses. The cycle threshold in each assay was set at a level where the exponential increase in amplicon abundance was approximately parallel between all samples. All mRNA abundance data were expressed relative to the amount of the constitutively expressed GAPDH. Conventional PCR was performed using Ampli-Taq Gold DNA polymerase (Applied Biosystems Ltd.). Pre-PCR cycle was 10 min at 95°C followed by 35 cycles of 95°C for 1 min, 56–60°C for 1 min and 72°C for 1 min followed by final extension 72°C for 10 min.

**Figure 3:**
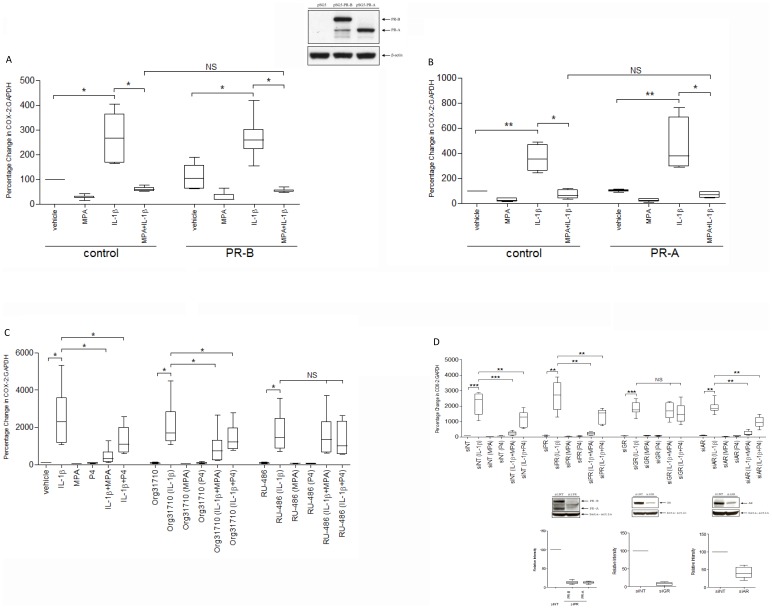
Effect of PR modulation on progesterone inhibition of IL-1β-driven COX-2 expression. **A and B,** Myometrial cells were transfected with PRB or PRA. SG5 was used as control. MPA and IL-1β, either alone or in combination were added 48 h after transfection and the cells were incubated for another 6 h. mRNA was then extracted, and the COX-2 mRNA levels were measured using qPCR. Data are expressed as median, 25^th^ and 75^th^ percentiles and range, and were analysed using Wilcoxon matched pairs test. *indicates a significant difference of *p*<0.05 and **, of *P*<0.01; NS indicates no statistic significant difference (n = 6). **C,** Myometrial cells were pre-incubated with Org31710 (1 µM) or Ru486 (1 µM) for 2 h before being exposed to different stimuli, IL-1β, MPA and progesterone, either alone or in combination. mRNA was then extracted, and the COX-2 mRNA levels were measured using qPCR. Data are expressed as median, 25^th^ and 75^th^ percentiles and range, and were analysed using paired *t* test. *indicates a significant difference of *p*<0.05; NS indicates no statistic significant difference (n = 6). **D,** Myometrial cells were transfected with different siRNAs against PR (siPR), GR (siGR) and AR (siAR), respectively. Non-targeting siRNA (siNT) was used as control. After transfection, cells were incubated for 96 h before being exposed to different stimuli, IL-1β, MPA and progesterone, either alone or in combination. mRNA was then extracted, and the COX-2 mRNA levels were measured using qPCR. Data are expressed as median, 25^th^ and 75^th^ percentiles and range, and were analysed using paired *t* test. **indicates a significant difference of *p*<0.01 and ***, of *P*<0.001; NS indicates no statistic significant difference (n = 6).

**Figure 4:**
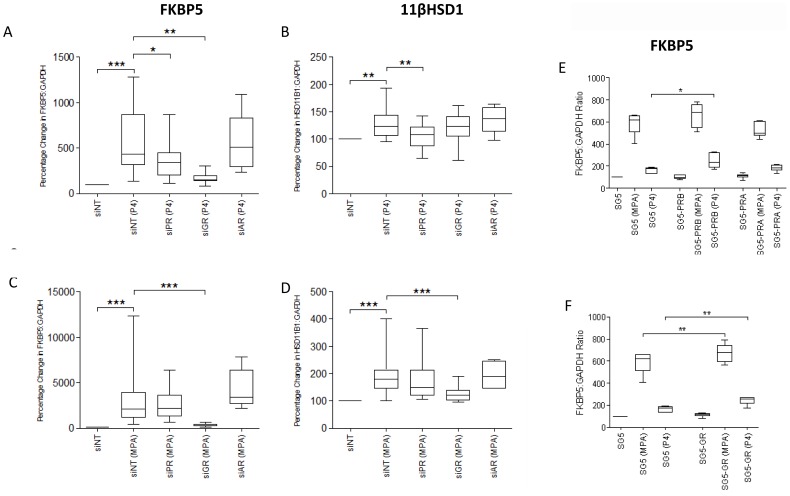
Effect of NR knockdown and over-expression on progesterone-responsive gene expression. **A–D,** Myometrial cells were transfected with different siRNAs against PR (siPR), GR (siGR) and AR (siAR), respectively. Non-targeting siRNA (siNT) was used as control. After transfection, cells were incubated for 96 h before being exposed to MPA or progesterone. **E&F,** Myometrial cells were transiently co-transfected with progesterone receptor B (PRB, Fig. 4E) or glucocorticoid receptor (GR, Fig. 4F). SG5 was used as control. mRNA was then extracted, and the mRNA levels of FKBP5 and HSD11β1 were measured using qPCR. Data are expressed as median, 25^th^ and 75^th^ percentiles and range, and were analysed using Wilcoxon matched pairs test. *indicates a significant difference of *p*<0.05, **, of *P*<0.01, and ***, of *P*<0.001 (n = 6–12).

### Western Analysis

Protein samples were prepared from monolayer myometrial cells by being lysed in Cell Lysis Buffer (New England Biolabs, Hitchin, SG4 0TY). The supernatant was separated from cell debris by centrifugation at 13,000×g for 20 min at 4°C. Protein concentrations were determined by Protein assay (Bio-Rad Laboratories Ltd., Hemel Hempsted, HP2 7DX) and bovine serum albumin (BSA) reference standards. Samples were then aliquot and stored at −80°C. Electrophoresis was carried out on 20 µg aliquots of protein samples that were denatured by adding NuPAGE loading Buffer (Invitrogen) and heating for 10 min at 70°C.

Western blotting was performed following an electrophoretic transfer onto a Hybond ECL nitrocellulose membrane (GE Healthcare, Slough, SL1 4ER). Membranes were blocked in blocking buffer (1×TBS and 5% milk) for 1 h at room temperature, washed in 1×TBS and hybridized with the primary antibody [antibody for COX-2 (SC-1745), NFκB p65 (SC-8008) and GR (SC-1003) used at 1∶1000, Santa Cruz Biotechnology Inc., Santa Cruz, Ca 95060; antibody for phospho-NFκB p65 (Ser536) used at 1∶1000, Cell signalling (3031), antibody for PR used at 1∶500, Leica (NCL-PGR-312); antibody for AR used at 1∶500, Dako (M3562); antibody for Halo tag used at 1∶1000, Promega (G9281); antibody for HSD11β1 used at 1∶1000, Abcam (ab83522); antibody for GRIP-1 used at 1∶1000, Millipore (07–1799); antibody for TATA bind protein (TBP) used at 1∶2000, Abcam (ab818); antibody for α-tubulin used at 1∶5000, Santa Cruz (SC-8035)] over night at 4°C. Membranes were washed again and then incubated with corresponding secondary antibody (New England Biolabs) at a dilution of 1∶2000 for 2 h at room temperature. ECL Plus (GE Healthcare Life Sciences) was used for detection. Protein band size was determined using Novex Sharp Pre-stained Protein Standard (Invitrogen).

### Co-immunoprecipitation (co-IP)

All immunoprecipitation procedures were performed at 4°C. Cells were harvested and washed twice with ice-cold PBS. Cells were lysed in lysis buffer [1% NP40, 150 mM NaCl, 50 mM Tris-HCl (pH 7.4), 1 mM EDTA and 10%Glycerol]. The lysate was pre-cleared by protein G-Agarose beads before the incubation with the antibody against the protein of interest (antibody for Strep tag, Qiagen; antibody for GR, Abnova) or pre-immune IgG of the same species 1–2 h with rotation. 10% of the lysate was kept as input. This lysate/antibody mixture was subsequently incubated with protein G-Agarose beads for 1 h with rotation. The beads were pelleted by centrifugation at 1000×g for 30 sec and washed four times in IP buffer [1% NP40, 150 mM NaCl, 50 mM Tris-HCl (pH 7.4) and 1 mM EDTA]. The protein–antibody complexes that were released from beads by adding the loading buffer and heating at 70°C for 10 min were subjected to Western blot analysis after separation by SDS–PAGE.

### Statistical Analysis

All data were initially tested for normality using a Kolmogorov-Smirnoff test. Normally distributed data were analysed using a Student’s *t* test for 2 groups and an analysis of variance (ANOVA) followed by a Dunnett or Bonferroni post hoc test for 3 groups or more. Data that were not normally distributed were analysed using a Wilcoxon matched pairs test for paired data and when comparing 3 groups or more a Friedman’s Test, with a Dunn’s Multiple Comparisons post hoc test. p<0.05 was considered statistically significant.

## Results

### IL-1β Represses Progesterone Action via NFκB

Using myometrial cells transfected with a progesterone reporter construct (PRE) we found that the overexpression of PRB in the presence of MPA increased PRE activation and this was repressed by IL-1β (p<0.05; Fig. 1A). Overexpression of p65 alone repressed MPA/PRB-driven PRE activity and this effect was more marked in the presence of IL-1β (both p<0.05; Fig. 1A). Knock down of p65 inhibited the ability of IL-1β to repress MPA/PRB activation of the PRE (Fig. 1B). This repression was evident in the smaller increase seen in FKBP5 mRNA expression in response to progesterone in IL-1β treated cells (Fig. 1C & S2). These data suggest that IL-1β-repression of progesterone/PR activity is mediated via NFκB.

### PRB Inhibits NFκB Activity

Conversely, overexpression of PRB alone inhibited p65-driven activation of an NFκB reporter construct (*p*<0.01) and this effect was enhanced by MPA (*p*<0.01; Fig. 2A). Similarly, MPA and progesterone repress IL-1β-driven COX-2 mRNA expression (Fig. 2B). The mutual repression is possibly explained by the ability of PRB and p65 to bind to each other as shown by the IP of halo-tagged PRB (Fig. 2C) and the increased binding of activated p65 to PRB in IL-1β-stimulated cells without overexpression of either PRB or p65 in a TF:TF array (unpublished data). These results supported the idea that PR interacts with p65 to inhibit NFκB transcriptional activity.

### Effect of PR Modulation on Progesterone Inhibition of IL-1β-driven COX-2 Expression

When we over expressed either PRB or PRA, neither enhanced the ability of MPA to repress IL-1β-driven COX-2 mRNA expression (Fig. 3A&B). Furthermore, the progesterone specific antagonist, Org-31710, did not block this effect although it reduced progesterone’s effects on its responsive genes (Fig. S3). Only Ru486, which inhibits both progesterone and glucocorticoid (GC) activity, was able to reverse the MPA and progesterone inhibition of IL-1β-driven COX-2 mRNA expression (Fig. 3C). Similarly when we knocked down PR and AR, this had no effect on either MPA or progesterone inhibition of IL-1β, only GR knock down reversed their ability to block IL-1β-driven COX-2 mRNA and protein expression (Fig. 3D; unpublished observation). The knockdown of each nuclear receptor was specific and did not reduce the level of other receptors (Fig. S4). To exclude the possibility that the action of progesterone was mediated though increased 11βHSD type 1 expression and the consequent increased conversion of cortisone to cortisol, we knocked down 11βHSD type 1 expression, but this had no effect on MPA/progesterone inhibition of IL-1β activity (Fig. S5). Further, the cortisol concentration was measured in the cell culture medium after incubation with all the different stimuli shown in Fig. 3D and no cortisol was detected. We then confirmed that GC acts exclusively via GR and not PR to repress IL-1β-driven COX-2 expression (Fig. S6). Overall, these data show that progesterone and MPA act via GR to inhibit the IL-1β-driven COX-2 expression.

### Progesterone Acts via both PR and GR to Drive Gene Expression

Progesterone increased FKBP5 mRNA expression via a combination of PR and GR, but increased 11βHSD mRNA via PR alone (Fig. 4A&B). Consistent with this, the effect of progesterone on FKBP5 was enhanced by both PRB and GR overexpression (Fig. 4E&F). In contrast MPA acted via GR exclusively to increase both FKBP5 and 11βHSD mRNA (Fig. 4C&D) and GR overexpression enhanced the MPA-induced FKBP5 expression (Fig. 4E&F).

## Discussion

These data suggest that progesterone inhibition of IL-1β-driven COX-2 expression in myometrial cells is mediated via a GR and not PR, but progesterone acts via both PR and GR to increase gene expression. However, the ability of IL-1β to inhibit MPA/PRB driven PRE activation and gene expression is probably mediated through an inhibition of PR action by p65.

The first study to identify a mutual inhibition between PR and NFκB found that this effect was independent of PR isoform, reporter construct, or cell type used and demonstrated a direct interaction between PR and p65 *in vitro* [13]. In addition, they found that TNFα treatment repressed PR activity, and that conversely PR repressed TNFα-induced NFκB activation [13]. These findings were replicated in primary amnion cells and in this study we found a similar response in primary myometrial cells, but in addition we confirmed that the IL-1β effects on PRB activity were mediated via p65 (14). We also confirmed that p65 and PRB interacted when we overexpressed halo-tagged PRB, but found that p65 did not bind to halo-tagged PRA. However, importantly, using a TF:TF array we were also able to show that activated p65 bound to PR in myometrial cells without overexpression of either p65 or PR, showing that this interaction can occur in the natural state. These data were consistent with the mutual inhibition identified by Kalkhoven et al [13] and consequently we expected when we increased PRB levels by transient transfection that we would see an enhanced inhibition of IL-1β-driven COX-2 expression. However, over-expression of PRB (or of PRA) had no effect on either progesterone or MPA inhibition of IL-1β activity. We then used the specific PR antagonist Org 31710 to block progesterone effects, but this agent had no effect, in contrast the PR/GR antagonist Ru486, reduced the inhibitory effect of both MPA and progesterone. Org31710 has a similar affinity for PR as Ru486, but a 30-fold lower affinity for GR [19], suggesting that neither progesterone nor MPA were acting via PR, rather the effect of Ru486 suggested that their action was mediated via GR. Consequently we knocked down the expression of PR, GR and AR and confirmed that both progesterone and MPA were acting via GR. However, since we had previously observed that progesterone enhanced the expression of 11βHSD1 in myometrial cells [11], it was possible that progesterone’s GR dependency was mediated via enhanced expression of 11βHSD1 and the consequent increase in conversion of cortisone to cortisol. However, no cortisol was detected in the cell culture medium, making it more likely that progesterone and MPA are acting directly via GR to mediate their effects on IL-1β driven COX-2 expression. Indeed, progesterone has been shown to act via GR to inhibit 20α-hydroxysteroid dehydrogenase in the rat corpus luteum [20] and to down-regulate the TLR4-mediated activation of macrophages [21]. However, the evidence in these papers was based on the absence of detectable PR and the ability of Ru486 to block the progesterone effects [20] and the inability of a specific PR agonist to down-regulate TLR4 actions [21]. In contrast, we have used the Ru486, an inhibitor of both PR and GR, compared to the effects of Org31710, a specific PR antagonist, supported by knock-down of PR and GR to prove that progesterone acts via GR to inhibit IL-1β-driven COX-2 expression. We also showed that progesterone acts via both PR and GR to up-regulate FKBP5 and via PR to up-regulate 11βHSD type 1 mRNA expression in myometrial cells. In contrast, the effects of MPA were mediated exclusively via GR. Further, although MPA has strong progestogenic and glucocorticoid effects, whether it, and indeed progesterone, acts via PR or GR to modulate gene expression may ultimately be determined by which co-activators or co-repressors are recruited to the transcription complex.

MPA was more potent than progesterone in the suppression of IL-1β-driven COX-2 expression, suggesting that it might be more effective than progesterone in the prevention of PTL. Indeed, in the original Liggins paper, when betamethasone was administered to mature the fetal lungs, the mean latency from treatment to delivery tended to be longer in the betamethasone group than in controls [22]. However, other studies have not shown any prolongation [23], still others actually an increase in uterine activity [24] and in the case of single vs. repeated antenatal steroid administration a shorter interval to delivery in the repeated steroid group [25]. Overall, these data suggest that the more potent glucocorticoid actions of MPA may not confer any clinical advantage over progesterone in the prevention of PTL.

These data show that while p65 does repress PR activity, progesterone acts via GR to repress IL-1β-induced gene expression. Thus, only half of the proposed mutual inhibition between PR and NFκB appears to be active in myometrial cells. Nevertheless, the ability of NFκB to repress PR may be sufficient to explain the withdrawal of progesterone action with the onset of human labour.

## Supporting Information

Figure S1 **The opitimizition of the concentration of MPA and P4.** Myometrial cells were exposed to different stimuli, IL-1β (5 ng/µl), MPA (1 µM, 10 µM and 100 µM) and P4 (1 µM, 10 µM and 100 µM), either alone or in combination. mRNA was then extracted, and the COX-2 mRNA levels were measured using qPCR. Data are expressed as mean±SEM (n = 3).
(TIF)

Figure S2 **IL-1β represses progesterone-responsive gene expression.** Myometrial cells were exposed to different stimuli, IL-1β, MPA and progesterone, either alone or in combination. mRNA was then extracted, and the FKBP5 mRNA levels were measured using qPCR. Data are expressed as median, 25^th^ and 75^th^ percentiles and range, and were analysed using Wilcoxon matched pairs test. ***indicates a significant difference of *P*<0.001 (n = 12).
(TIF)

Figure S3 **Effect of progesterone specific antagonist on progesterone-responsive genes.** Myometrial cells were pre-incubated with Org31710 (1 µM) for 2 h before being exposed to progesterone. mRNA was then extracted, and the mRNA levels of FKBP5, HSD11β1 and IL-1β were measured using qPCR. Data are expressed as median, 25^th^ and 75^th^ percentiles and range, and were analysed using paired *t* test. *indicates a significant difference of *p*<0.05 (n = 6).
(TIF)

Figure S4 **The specificity of hormone receptors knockdown.** Myometrial cells were transfected with different siRNAs against PR (siPR), GR (siGR) and AR (siAR) for 96 h, respectively. Non-targeting siRNA (siNT) was used as control.
(TIF)

Figure S5 **Effect of HSD11β1 knockdown on IL-1β-driven COX-2 expression.** Myometrial cells were transfected with siRNA against HSD11β1 (siHSD11β1). siNT was used as control. After transfection, cells were incubated for 96 h before being exposed to different stimuli, IL-1β, MPA, progesterone and dexamethasone, either alone or in combination. mRNA was then extracted, and the COX-2 mRNA levels were measured using qPCR. Data are expressed as mean±SEM (n = 3).
(TIF)

Figure S6 **GR mediates GC’s effect on COX-2 expression.** Myometrial cells were transfected with different siRNAs against PR and GR. siNT was used as control. After transfection, cells were incubated for 96 h before being exposed to different stimuli, IL-1β and Dex, either alone or in combination. mRNA was then extracted, and the COX-2 mRNA levels were measured using qPCR. Data are expressed as median, 25^th^ and 75^th^ percentiles and range, and were analysed using Wilcoxon matched pairs test. *indicates a significant difference of *p*<0.05 and # of *P*<0.05 between samples exposed to both IL-1β and Dex with or without GR knockdown (n = 6).
(TIF)
