# Supplementary figures and images for: Progesterone Acts via the Nuclear Glucocorticoid Receptor to Suppress IL-1β-Induced COX-2 Expression in Human Term Myometrial Cells
Source: PLoS One. 2012 Nov 28;7(11):e50167. doi: 10.1371/journal.pone.0050167 (PMC3509141; doi:10.1371/journal.pone.0050167)

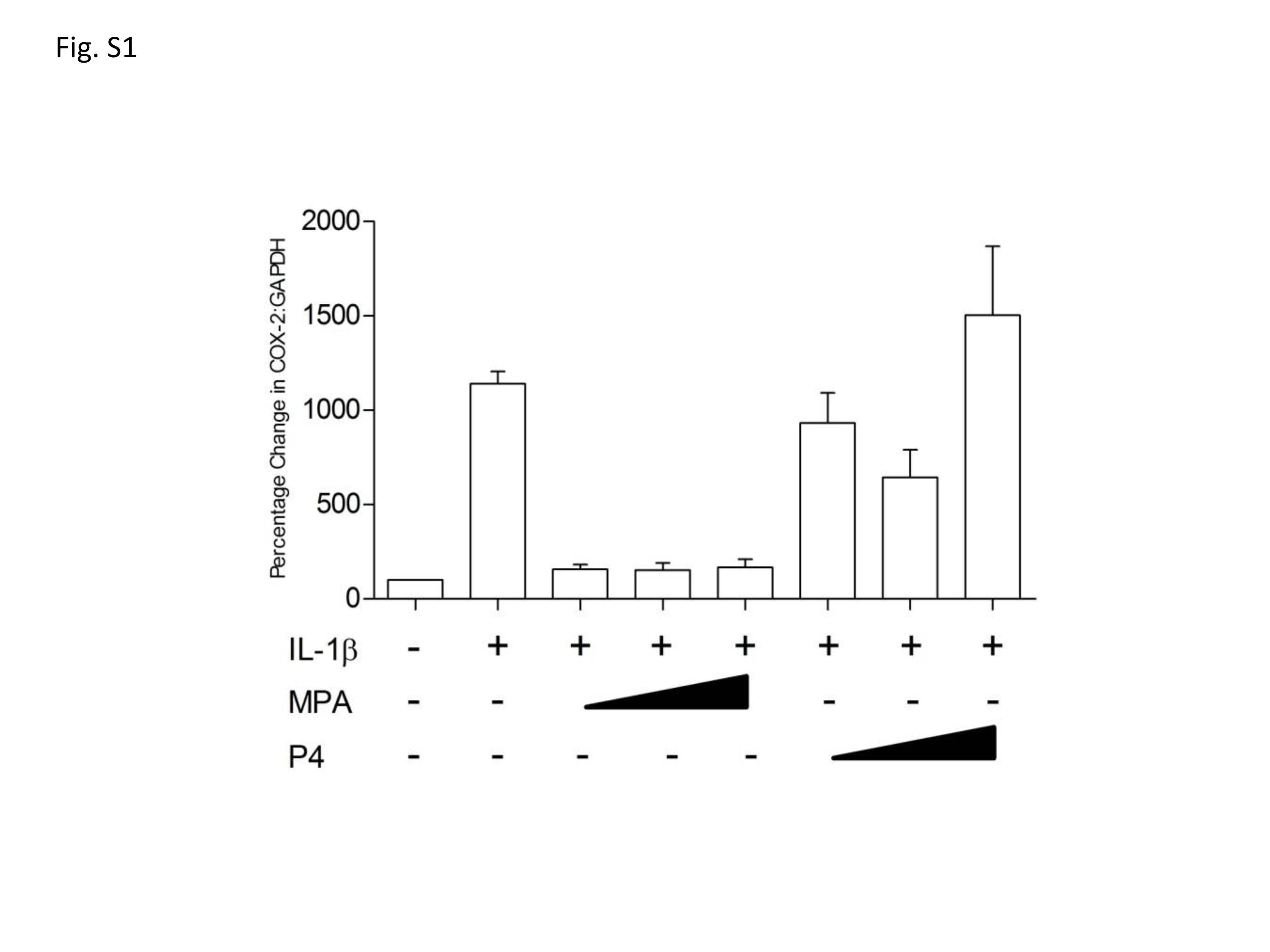

Supplement: Figure S1 — The opitimizition of the concentration of MPA and P4. Myometrial cells were exposed to different stimuli, IL-1β (5 ng/µl), MPA (1 µM, 10 µM and 100 µM) and P4 (1 µM, 10 µM and 100 µM), either alone or in combination. mRNA was then extracted, and the COX-2 mRNA levels were measured using qPCR. Data are expressed as mean±SEM (n = 3). (TIF) [file pone.0050167.s001.tif]

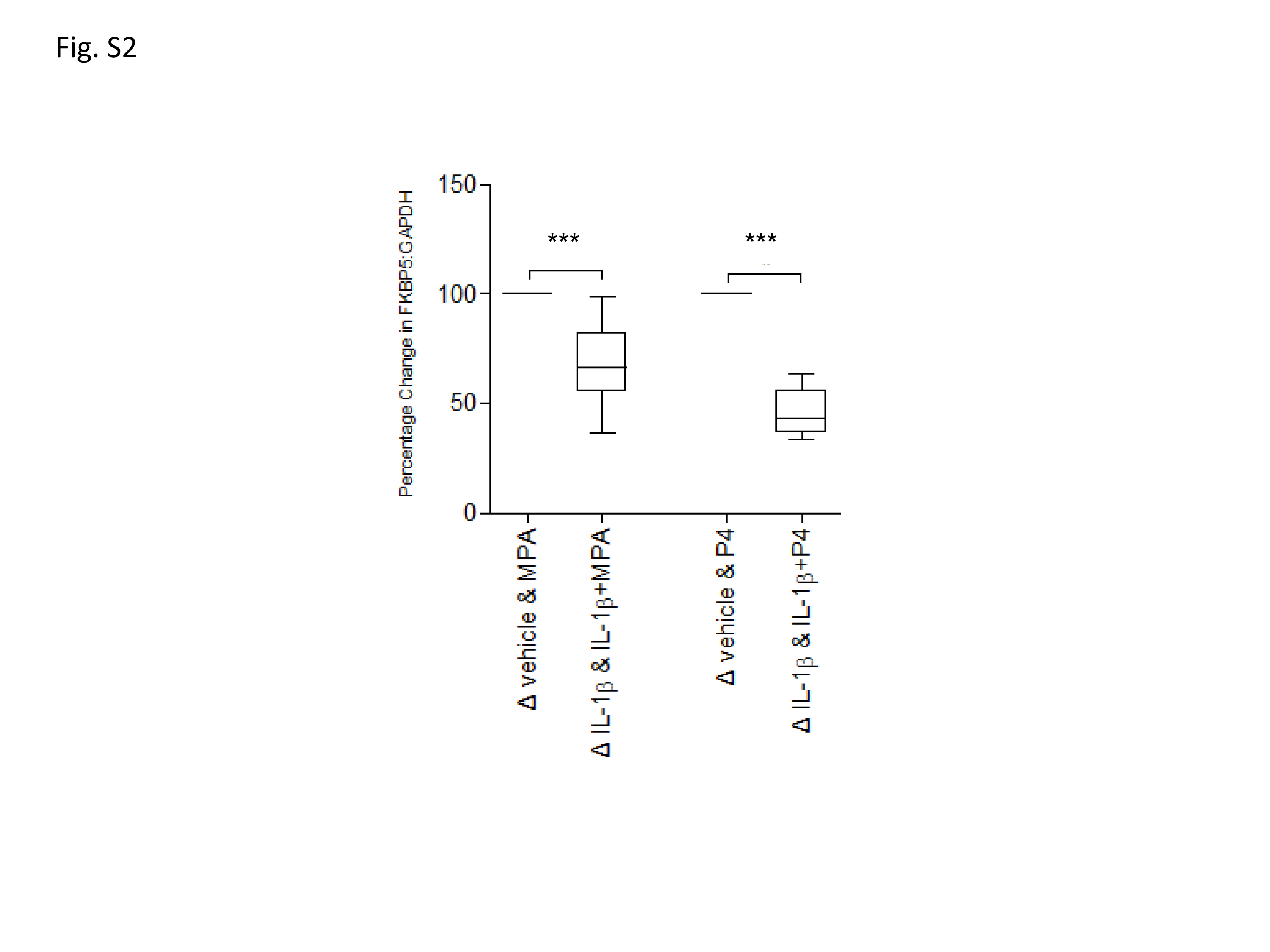

Supplement: Figure S2 — IL-1β represses progesterone-responsive gene expression. Myometrial cells were exposed to different stimuli, IL-1β, MPA and progesterone, either alone or in combination. mRNA was then extracted, and the FKBP5 mRNA levels were measured using qPCR. Data are expressed as median, 25th and 75th percentiles and range, and were analysed using Wilcoxon matched pairs test. ***indicates a significant difference of P<0.001 (n = 12). (TIF) [file pone.0050167.s002.tif]

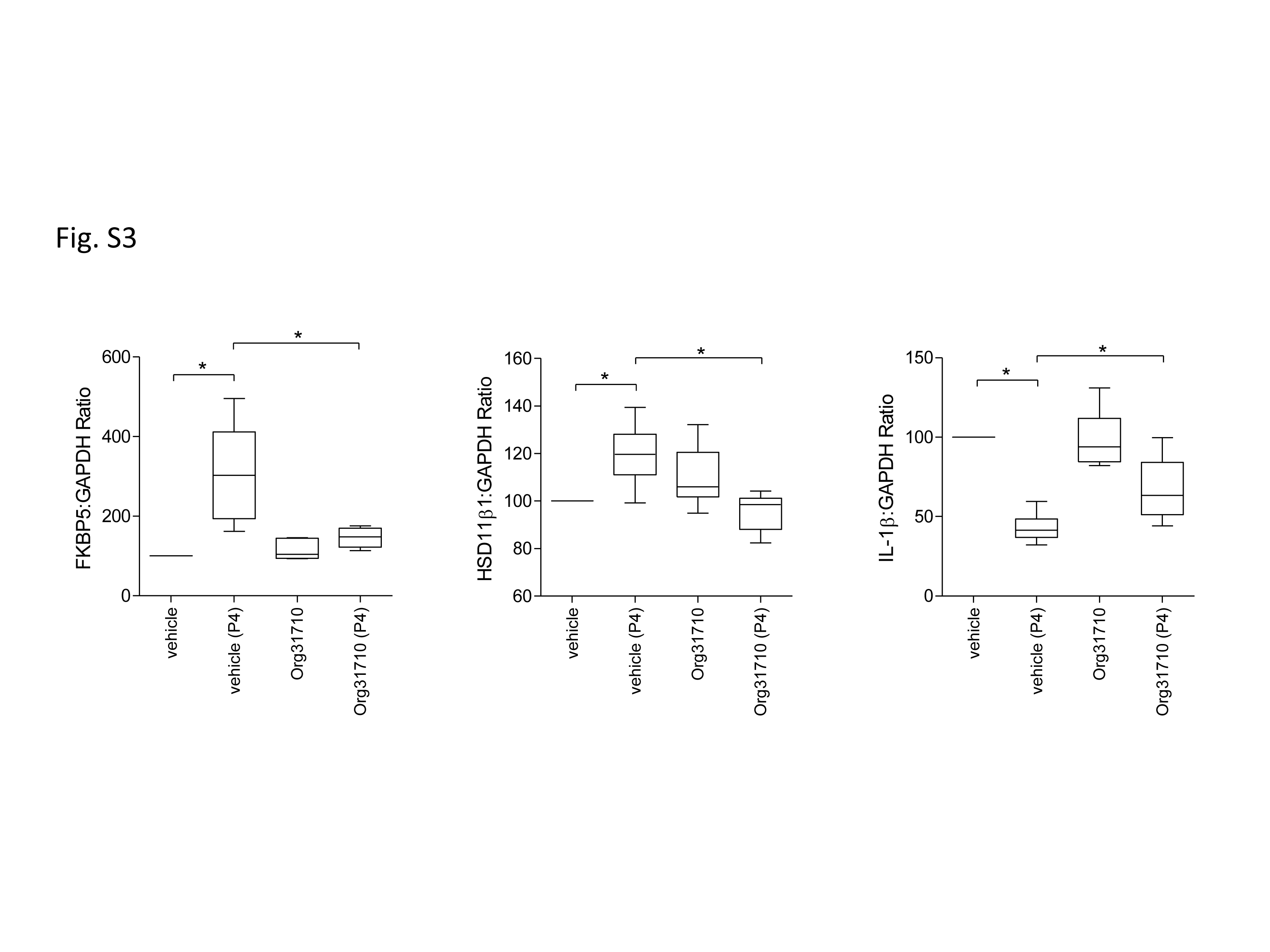

Supplement: Figure S3 — Effect of progesterone specific antagonist on progesterone-responsive genes. Myometrial cells were pre-incubated with Org31710 (1 µM) for 2 h before being exposed to progesterone. mRNA was then extracted, and the mRNA levels of FKBP5, HSD11β1 and IL-1β were measured using qPCR. Data are expressed as median, 25th and 75th percentiles and range, and were analysed using paired t test. *indicates a significant difference of p<0.05 (n = 6). (TIF) [file pone.0050167.s003.tif]

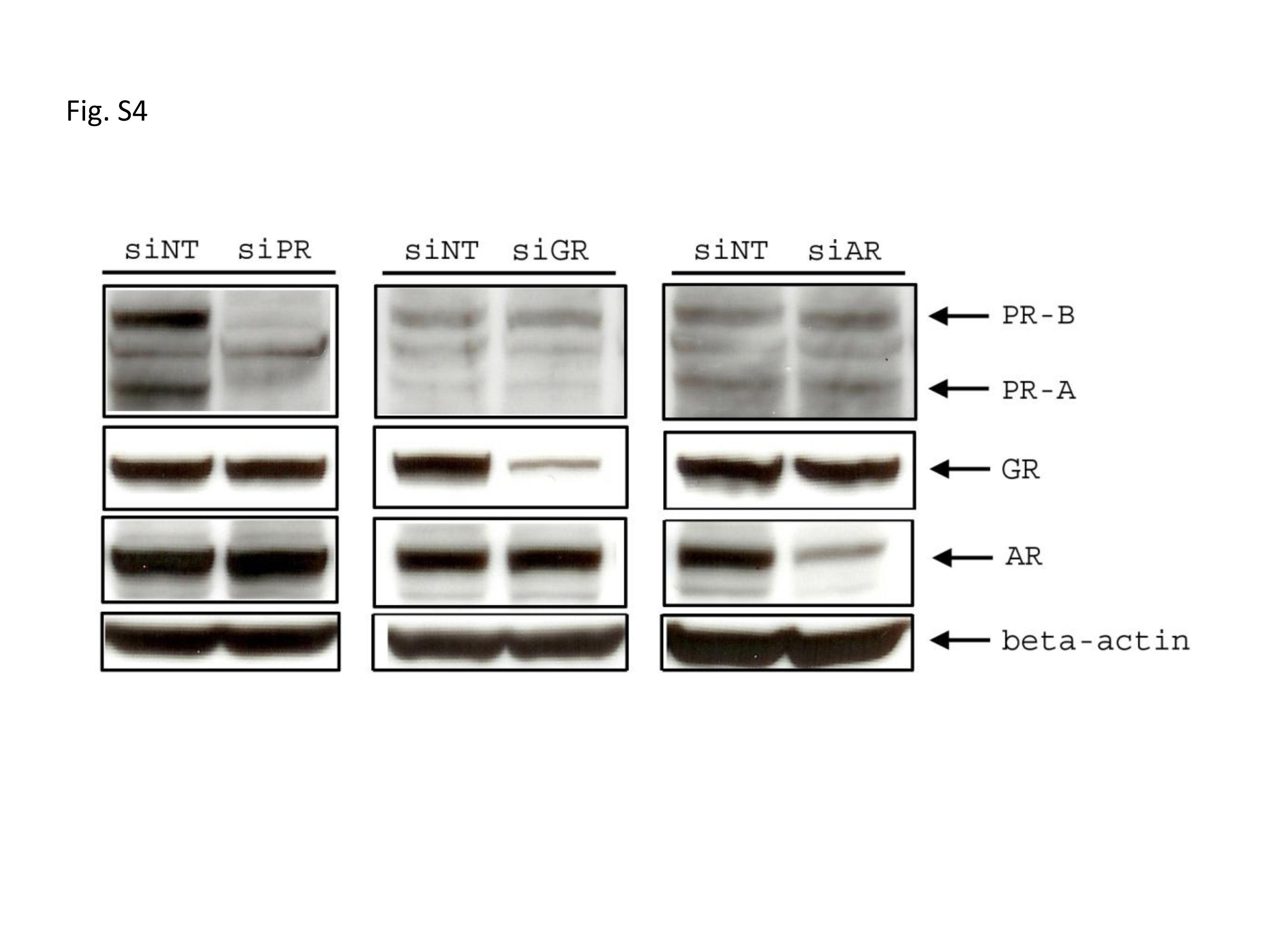

Supplement: Figure S4 — The specificity of hormone receptors knockdown. Myometrial cells were transfected with different siRNAs against PR (siPR), GR (siGR) and AR (siAR) for 96 h, respectively. Non-targeting siRNA (siNT) was used as control. (TIF) [file pone.0050167.s004.tif]

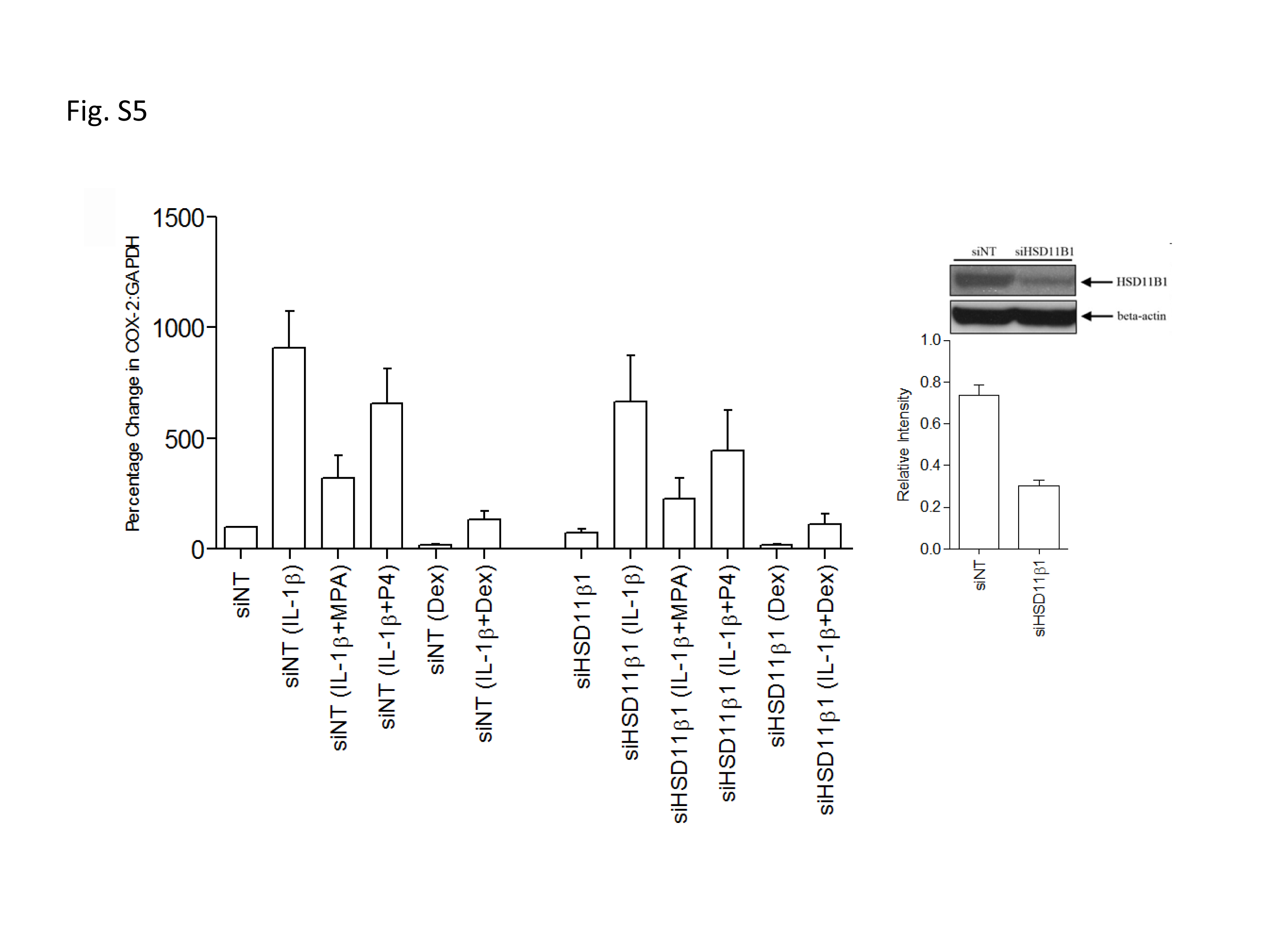

Supplement: Figure S5 — Effect of HSD11β1 knockdown on IL-1β-driven COX-2 expression. Myometrial cells were transfected with siRNA against HSD11β1 (siHSD11β1). siNT was used as control. After transfection, cells were incubated for 96 h before being exposed to different stimuli, IL-1β, MPA, progesterone and dexamethasone, either alone or in combination. mRNA was then extracted, and the COX-2 mRNA levels were measured using qPCR. Data are expressed as mean±SEM (n = 3). (TIF) [file pone.0050167.s005.tif]

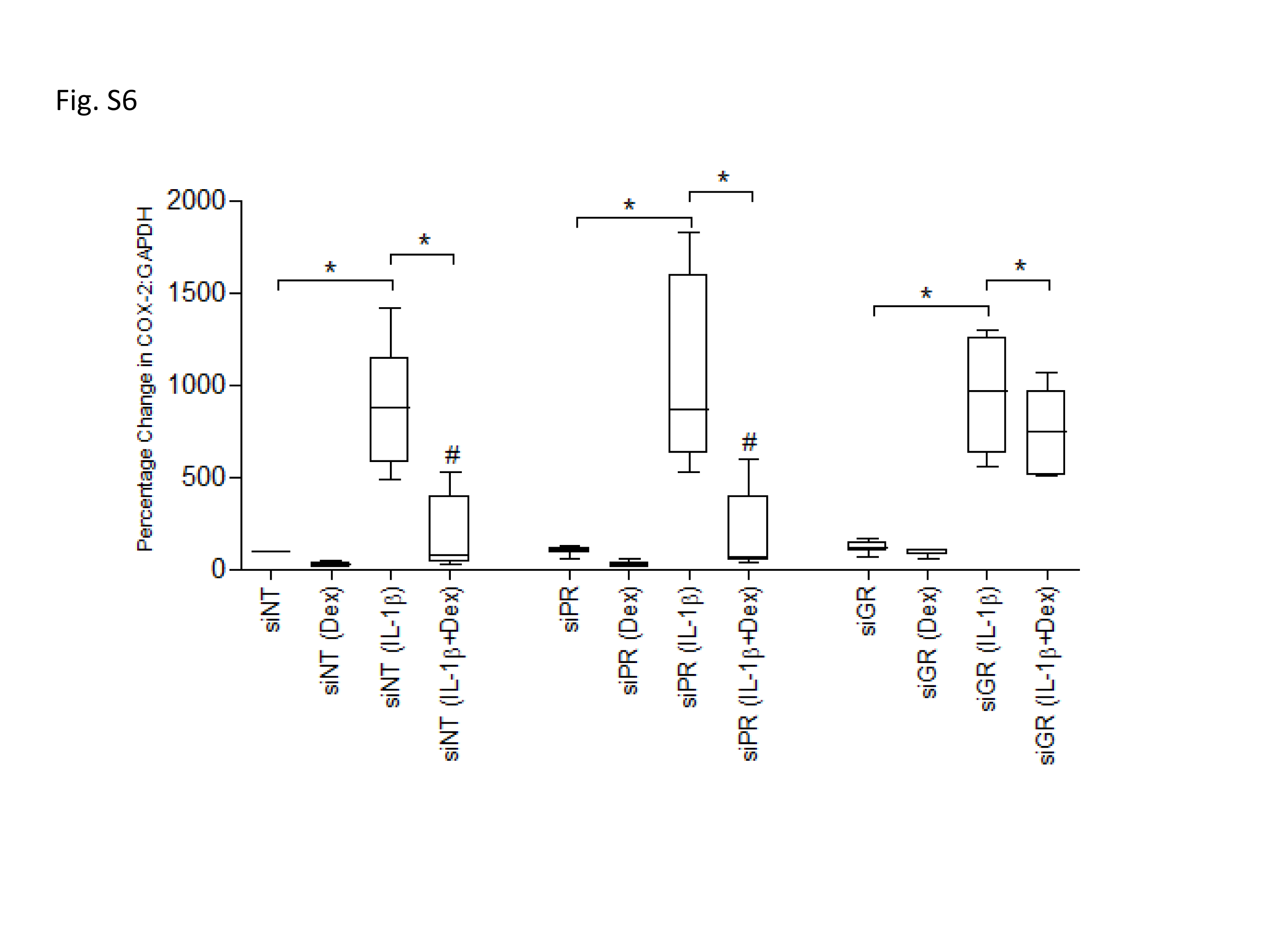

Supplement: Figure S6 — GR mediates GC’s effect on COX-2 expression. Myometrial cells were transfected with different siRNAs against PR and GR. siNT was used as control. After transfection, cells were incubated for 96 h before being exposed to different stimuli, IL-1β and Dex, either alone or in combination. mRNA was then extracted, and the COX-2 mRNA levels were measured using qPCR. Data are expressed as median, 25th and 75th percentiles and range, and were analysed using Wilcoxon matched pairs test. *indicates a significant difference of p<0.05 and # of P<0.05 between samples exposed to both IL-1β and Dex with or without GR knockdown (n = 6). (TIF) [file pone.0050167.s006.tif]
